# Supplementary material for: The genome sequence of Adonis annua L., 1753 (Ranunculales: Ranunculaceae)
Source: Wellcome Open Res. 2026 May 26;11:302. [Version 1] doi: 10.12688/wellcomeopenres.26636.1 (PMC13316066; doi:10.12688/wellcomeopenres.26636.1)
Supplement: Peer review history

# Reviewer report: Reviewer response for version 1

**DOI:** 10.21956/wellcomeopenres.29332.r158007

**Published:** 2026-07-03

**Authors:** Roberto Biello

**Affiliations:** 1 University of Copenhagen Globe Institute, Copenhagen, Capital Region of Denmark, Denmark

The authors present a high-quality genome assembly for pheasant’s-eye, *Adonis annua*, using PacBio HiFi and Hi-C sequencing. This represents a valuable genomic resource for an endangered arable plant species in Britain, with potential relevance for conservation genomics, population studies, and understanding genome evolution in Ranunculaceae. The assembly is highly contiguous, with most of haplotype 1 assigned to 16 chromosomal-level scaffolds, and the reported QV and k-mer completeness values are good. The methods are clearly described, and the associated data are publicly available. A few points require clarification.

 Comments:

 - The relationship between the flow cytometry estimate, the GenomeScope estimate, and the very large assembled haplotypes should be clarified, particularly in light of the reported cytological variation and possible tetraploidy in *A. annua*.

 - The authors state that GenomeScope2 estimated a haploid genome size of 7,123.48 Mb, heterozygosity of 13.04%, and repeat content of 46.46%, but these values do not correspond to the real values from Figure 2. The authors should ensure that the figure, legend, or accompanying text displays these key GenomeScope results clearly.

 - The BUSCO results show very high completeness but an unusually high proportion of duplicated genes. The authors should explicitly discuss whether this reflects the biology of the species, polyploidy, or residual assembly redundancy.

 - The manuscript reports 0.00 Gb and 0.00 million Hi-C reads, yet Hi-C data were used for scaffolding and chromosome validation. This appears to be an error and should be corrected.

Are sufficient details of methods and materials provided to allow replication by others?

Partly

Is the rationale for creating the dataset(s) clearly described?

Yes

Are the datasets clearly presented in a useable and accessible format?

Yes

Are the protocols appropriate and is the work technically sound?

Partly

Reviewer Expertise:

Population genomics, Genome assembly

I confirm that I have read this submission and believe that I have an appropriate level of expertise to confirm that it is of an acceptable scientific standard, however I have significant reservations, as outlined above.

# Reviewer report: Reviewer response for version 1

**DOI:** 10.21956/wellcomeopenres.29332.r158001

**Published:** 2026-06-29

**Authors:** Liangsheng Zhang

**Affiliations:** 1 Zhejiang University, Hangzhou, Zhejiang, China

**Adonis annua** has a large genome, and a high-quality genome was obtained. My only suggestion is that Haplotype 2 could be scaffolded onto chromosomes using Haplotype 1 as a reference with RagTag, so that Haplotype 2 can also be produced in a chromosome-scale version.

**Haplotype 1** serves as the reference assembly, already anchored to chromosomes with well-defined genomic coordinates.

**Haplotype 2**, while structurally similar, likely contains allele-specific variations including SNPs, indels, and structural differences (e.g., inversions, translocations, or copy number variations) that reflect heterozygosity in the diploid genome. Since **Adonis annua** has a large genome, these haplotype-specific differences may span substantial regions. Using RagTag to scaffold Haplotype 2 against Haplotype 1 would preserve these differences while providing a syntenic, chromosome-scale framework for downstream comparative and phasing analyses.

Are sufficient details of methods and materials provided to allow replication by others?

Yes

Is the rationale for creating the dataset(s) clearly described?

Yes

Are the datasets clearly presented in a useable and accessible format?

Yes

Are the protocols appropriate and is the work technically sound?

Yes

Reviewer Expertise:

plant genomes

I confirm that I have read this submission and believe that I have an appropriate level of expertise to confirm that it is of an acceptable scientific standard.

# Reviewer report: Reviewer response for version 1

**DOI:** 10.21956/wellcomeopenres.29332.r156770

**Published:** 2026-06-17

**Authors:** Oliver Rupp

**Affiliations:** 1 Justus-Liebig-University, Giessengiessen, Germany

This article presents the assembly of the Ranunculaceae Adonis annua.

 The data creation and data analyses follow state-of-the-art methods for eukaryotic genome assemblies established at the Darwin Tree of Life Consortium, including PacBio Hifi and Hi-C sequencing. The assembly with hifiasm in Hi-C mode was followed by Hi-C scaffolding and manual curation.

 The assembly quality assessment using k-mer and orthology based methods like Merqury.FK or BUSCO showed a high-quality sequence for both haplotypes.

 The methods are clearly presented, and the quality of the data is shown.

 Overall, no major issues are to report.

Are sufficient details of methods and materials provided to allow replication by others?

Yes

Is the rationale for creating the dataset(s) clearly described?

Yes

Are the datasets clearly presented in a useable and accessible format?

Yes

Are the protocols appropriate and is the work technically sound?

Yes

Reviewer Expertise:

genome assembly

I confirm that I have read this submission and believe that I have an appropriate level of expertise to confirm that it is of an acceptable scientific standard.

# Reviewer report: Reviewer response for version 1

**DOI:** 10.21956/wellcomeopenres.29332.r158002

**Published:** 2026-06-16

**Authors:** Abdoallah Sharaf

**Affiliations:** 1 University of Konstanz, Konstanz, Germany

The manuscript presents a haplotype-resolved genome assembly for *Adonis annua* (Pheasant’s-eye), a large (~14 Gb) and complex genome from an Endangered UK archaeophyte, produced as part of the Darwin Tree of Life project. Haplotype 1 is scaffolded to chromosome level (14,092.46 Mb, 97.27% in 16 pseudomolecules), while haplotype 2 remains at scaffold level. Organelle genomes were also assembled. The work follows DToL protocols and provides useful data for Ranunculaceae and conservation genomics of arable annuals. High heterozygosity (13.04%) and repeat content (46.46%) are consistent with the species’ polyploid nature.

 However, several aspects of assembly quality, validation, and presentation require clarification and improvement before the manuscript can be considered a robust high-quality reference genome.

**Major points (requiring author response):**

1. **Noisy Hi-C contact map with extensive cross-chromosome signals** The Hi-C contact map (Figure 3) exhibits very high background noise and numerous off-diagonal/cross-chromosome interactions. This level of noise is not addressed in the text, despite 17 breaks and 30 joins during manual curation. **Recommendation:** Discuss the Hi-C data quality, any steps taken to reduce noise, and the resulting confidence in the 16 chromosomal pseudomolecules. High heterozygosity/polyploidy may contribute, but this should be explicitly explained.
2. **Unclassified contig groups in BlobToolKit plot potentially indicating contamination** The BlobToolKit blob plot (Figure 6) shows multiple distinct groups of contigs/scaffolds with no taxonomic assignment (unclassified / “no hit”). These could represent contaminants, novel sequences, or assembly artefacts, yet they are not discussed. **Recommendation:** Investigate and comment on these unclassified clusters in the Assembly quality assessment section. If they are potential contaminants, describe additional decontamination steps taken or why they were retained. Provide GC-coverage statistics or further BLAST results for these groups.
3. **Extreme BUSCO duplication rate and EBP classification** BUSCO (embryophyta_odb10) reports 99.4% completeness but with only 4.8% single-copy and 94.6% duplicated genes. While expected in a polyploid, this is exceptionally high. The claimed EBP rating of **6.C.Q60** appears optimistic given the duplication level and EBP preferences for >90% single-copy genes. **Recommendation:** Contextualize the results in light of the species’ known cytological variation (2n=16 vs 2n=32) and consider additional BUSCO runs against more specific lineages. Revise Table 4 accordingly with transparent justification.
4. **Large discrepancy between genome size estimates** Flow cytometry gave a 1C-value of 15.65 pg (≈15,300 Mb), while GenomeScope2 estimated ~7,123 Mb. The assembled haplotypes (~14 Gb each) align better with flow cytometry, but the ~2× difference is not adequately discussed. **Recommendation:** Present the flow cytometry histogram (with peak CVs) as a supplementary figure and explain the discrepancy in the context of polyploidy.
5. **DNA barcoding results not reported** Barcoding was performed per the DToL framework and compared to BOLD/GenBank, but no outcomes (BOLD IDs, accessions, similarity scores) are provided. **Recommendation:** Report the barcoding results explicitly to confirm taxonomic identity.

**Minor points:**

- Hi-C sequencing yielded substantial data (876.54 Gb), yet scaffolding challenges remain — a brief comment on Hi-C coverage or quality would be helpful.
- The high number of remaining scaffolds in haplotype 1 (7,365) after curation should be contextualized.
- Strengthen the biological/conservation discussion on how this genome may inform population genetics and management of this declining species.

**Overall assessment:**

 This is an ambitious and important genome project for a large polyploid plant of conservation concern. Contiguity and QV are reasonable given the genome complexity, but the noisy Hi-C map, unclassified contig groups in BTK, extreme BUSCO duplication, genome size discrepancy, and missing barcoding results need to be properly addressed and justified. Resolving these issues would markedly strengthen the manuscript as a reliable reference resource.

Are sufficient details of methods and materials provided to allow replication by others?

Partly

Is the rationale for creating the dataset(s) clearly described?

Yes

Are the datasets clearly presented in a useable and accessible format?

Yes

Are the protocols appropriate and is the work technically sound?

Partly

Reviewer Expertise:

Genomics, bioinformatics, plant genome assembly, polyploid genomes, and biodiversity research.

I confirm that I have read this submission and believe that I have an appropriate level of expertise to confirm that it is of an acceptable scientific standard, however I have significant reservations, as outlined above.
